# Double localisation secondaire cutanée et sinusienne révélant un adénocarcinome à cellules claires du rein: un cas avec revue de la littérature

**DOI:** 10.11604/pamj.2018.31.66.16721

**Published:** 2018-10-01

**Authors:** Eliane Ndounga, Jean Felix Peko, Jean Bernard Nkoua Mbon

**Affiliations:** 1 Service d’Oncologie Médicale, CHU de Brazzaville, République du Congo; 2 Service d’Anatomie Cytologie Pathologique, CHU de Brazzaville, République du Congo

**Keywords:** Cancer du rein, métastases, thérapies antigéniques, Kidney cancer, metastasis, antigen-based therapy

## Abstract

Situé au troisième rang des cancers urologiques, le cancer du rein métastase habituellement au niveau du poumon, des os et du foie. Nous rapportons ici le cas clinique de métastases cutanée et sinusienne ayant révélées un cancer du rein chez un sujet de sexe masculin âgé de 70 ans.

## Abstract (en)

Kidney cancer is the third most frequent urologic cancer. Metastases usually occur in the lung, the bone and the liver. We here report a clinical case of cutaneous and sinus metastases revealing kidney cancer in a 70 year old man.

## Introduction

Le cancer du rein représente 3% de l'ensemble des tumeurs malignes de l'adulte et se situe au troisième rang des cancers urologiques [1]. Les sièges métastatiques les plus fréquents de cancer sont habituellement pulmonaire, osseuse et hépatique [1]. En revanche, les métastases cutanées et oto-rhino-laryngologique (ORL) de ce cancer sont rares. Nous rapportons un cas de localisation secondaire au scalp et au sinus sphénoïdal ayant révélé un carcinome à cellules claires chez un patient de 70 ans alcoolo-tabagique.

## Patient et observation

**Figure 1:**
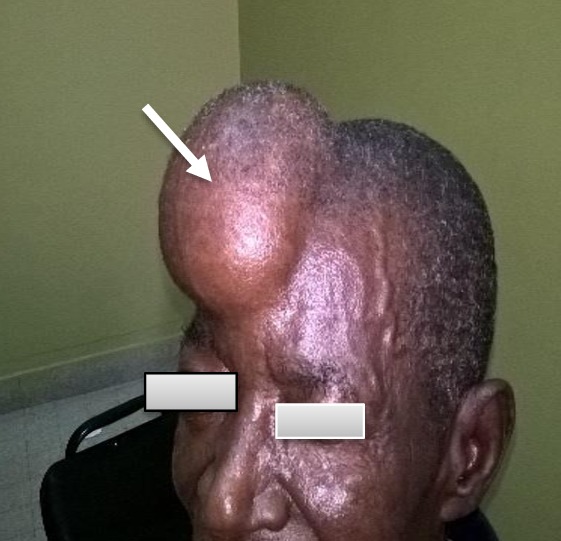
Volumineuse néoformation frontale latéralisée à droite, d’environ 14cm de grand axe avec circulation veineuse collatérale

Monsieur B T, âgé de 70 ans, alcoolo-tabagique depuis plus de 40 ans à raison de 33 paquet-année et 40g d'alcool par jour, a consulté dans notre service pour une néoformation du scalp d'évolution progressivement croissante depuis 18 mois associé à une altération progressive de l'état général. L'examen clinique à l'entrée a permis de mettre en évidence un amaigrissement avec un IMC à 17,54kg/m², une exophtalmie droite sans paralysie faciale, une volumineuse néoformation du scalp, latéralisée à droite mesurant 14cmx12cm de diamètre, indolore, fixée au deux plans (Figure 1). L'abdomen était souple, indolore, sans masse palpable; le toucher rectal était sans particularité. L'auscultation cardio-pulmonaire a retrouvé un syndrome de condensation pulmonaire bilatérale.

**Figure 2:**
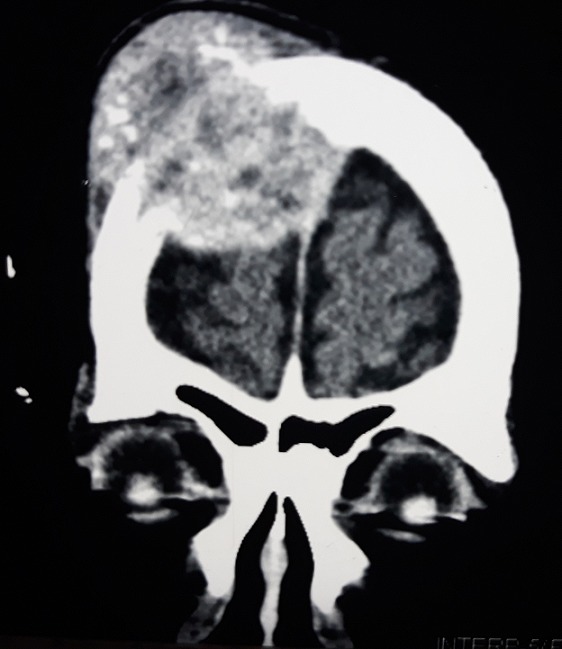
Lésion tissulaire lytique frontale hétérogène, fortement rehaussée après injection, mesurant environ 20cm de grand axe

**Figure 3:**
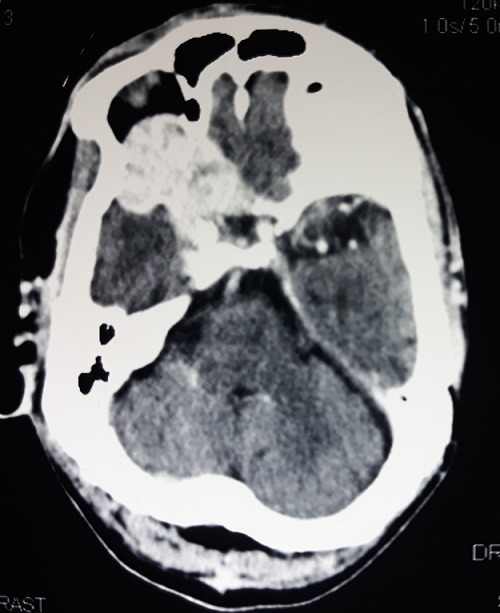
Lésion au niveau du sinus sphénoïdal, s’étendant à la cavité orbitaire et infiltrant le nerf optique et la graisse intra et extra-conique

**Figure 4:**
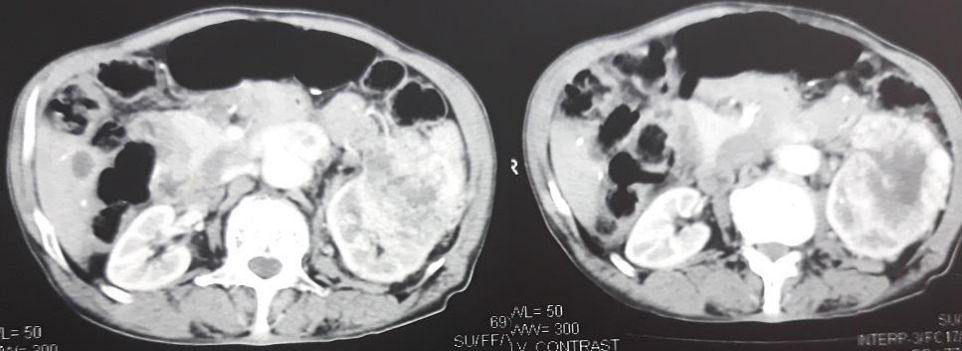
Micrographie 1 (HE) Gx200: prolifération tumorale d’aspect végétal, faite de cellules globuleuses claires de taille variable

Le scanner cérébral a permis de noter la présence d'une lésion tissulaire, lytique, frontale, latéralisée à droite, hétérogène, fortement rehaussée après injection de produit de contraste, mesurant environ 20cm de grand axeau contact de la faux du cerveau en dehors. Une autre lésion ayant les mêmes caractéristiques a été identifiée au niveau du sphénoïde, s'étendant à la cavité orbitaire, infiltrant le nerf optique, la graisse intra et extra conique, à l'origine d'une exophtalmie grade II. Cette lésion était au contact de la région basifrontale droite en dedans et de la région temporale interne en dehors (Figure 2 et Figure 3). L'examen anatomopathologique a objectivé une prolifération tumorale réalisant des lobules adossés faits de cellules claires d'aspect végétal; les atypies nucléaires modérées; mitoses rares et stroma endocrinoïde en faveur d'une métastase d'un adénocarcinome à cellules claires (Figure 4).

**Figure 5:**
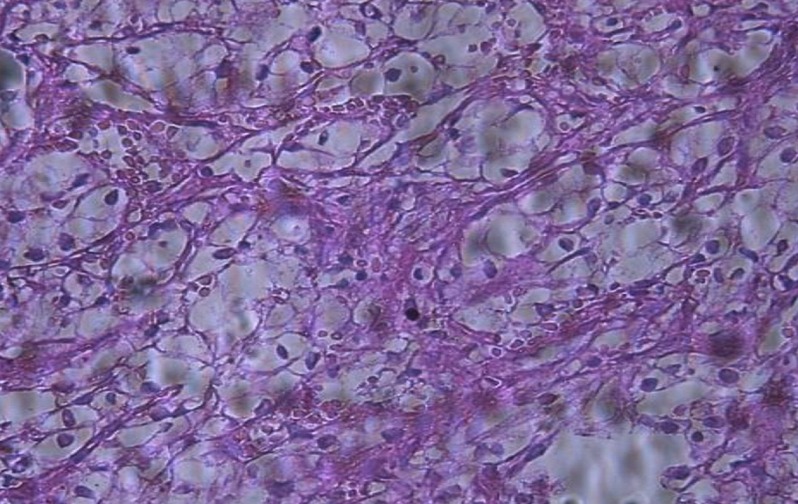
Volumineuse formation tumorale hétérogène aux dépens du rein gauche, avec de larges plages de nécrose, mesurant environ 18x13cm

Un scanner thoraco-abdominale a retrouvé une volumineuse formation tumorale hétérogène aux dépens du rein gauche, avec de larges plages de nécrose, mesurant environ 18x13mm de diamètres; la présence d'adénopathies nécrosées au niveau du hile, Il n'y a pas de dilatation des cavités pyélocalicielles et d'une lésion hypodense du segment VIII du foie; des opacités interstitielles des deux champs pulmonaires, la présence d'opacités excavées du lobe supérieur droit et nodulaires du lobe supérieur gauche à l'étage thoracique (Figure 5). Le bilan biologique a été sans particularité en dehors d'une hypocalcémie à 65mg/l (Normale: 80-110 mg/L). Le diagnostic retenu a été un adénocarcinome à cellules claires du rein avec métastases cutanée, sphénoïdale, pulmonaire et hépatique.

## Discussion

L'intérêt de cette observation est double. Il repose d'une part sur le mode de révélation du cancer rénal, et d'autre part sur cette double localisation métastatique exceptionnelle. En effet, les métastases cutanées du cancer du rein sont rares. Les cancers primitifs les plus souvent à l'origine de ces métastases sont ceux du poumon, du mélanome malin et des tumeurs de la cavité buccale [1]. Au niveau cutané, Le cuir chevelu et la face sont les sites majoritaires de ces métastases [2]. Le carcinome à cellules claires du rein représente 6% des cancers à l'origine des métastases cutanées [1]. Dans une étude rétrospective sur 12 ans ayant porté sur 306 patients opérés d'un cancer du rein, les métastases cutanées n'ont révélé le cancer du rein que chez un seul patient [2].

Ces métastases utilisent principalement la voie lymphatique associée à la voie veineuse comme mode de dissémination. Elles ne sont jamais uniques, comme cela a été le cas chez notre patient. La diffusion métastatique à d'autres sites métastatiques au moment du diagnostic explique la médiane de survie inférieure à 6 mois [1]. Au niveau de la tête et du cou, les métastases d'origine rénale représentent également 6% de l'ensemble des métastases du cancer du rein [1]. Au niveau oto-rhino-laryngologique (ORL), les trois sites métastatiques les plus fréquentes d'origine rénal sont les sinus paranasal et maxillaire, la thyroïde et les glandes salivaires [1]. Les localisations secondaires au sinus sphénoïdal sont rares; elle est unique dans plus de 50% des cas [3]. Différents mécanismes expliquent cette affinité particulière des cellules rénales pour les sinus propagation de micro-emboles artériels jusqu'aux microcapillaires constituant ces tissus, utilisation des plexus veineux prévertébraux [1,3].

Le traitement médical du cancer du rein métastatique repose, depuis 2006, sur les molécules anti-angiogéniques. Leur efficacité a été principalement évaluée en cas de carcinome rénal à cellules claires (5). Pour la chirurgie des métastases multiples, comme observé chez notre patient, peu de données sont disponibles. La résection chirurgicale pourrait permettre d'améliorer la survie [4]. Les patients porteurs de cancer du rein avec des métastases osseuses ont un mauvais pronostic. Le taux de survie à cinq ans pour les patients avec des métastases osseuses multiples, comme c'est le cas pour notre patient, est de 5 à 15% [5].

## Conclusion

Parmi les localisations secondaires inhabituelles du carcinome rénal à cellules claires, le scalp et le sinus sphénoïdal sont des sites métastatiques rares. Leur résection chirurgicale reste bénéfique sur la survie en association avec le traitement médical à base de thérapies antigéniques.

## Conflits d’intérêts

Les auteurs ne déclarent aucun conflit d'intérêts.
